# GP Surgeons’ Experiences of Training in British Columbia and Alberta: A Case Study of Enhanced Skills for Rural Primary Care providers

**Published:** 2012-03-31

**Authors:** Jude Kornelsen, Stuart Iglesias, Nancy Humber, Nadine Caron, Stefan Grzybowski

**Affiliations:** 1 Centre for Rural Health Research; 2 University of British Columbia; 3 Rural Family Physician; 4 University of Northern British Columbia; 5 General Surgeon

## Abstract

**Introduction:**

There has been a steady erosion of family physicians with enhanced surgical skills providing care for rural residents. This has been largely due to the lack of formal training avenues and continuing medical education (CME) opportunities afforded to those interested, and attrition of those currently practicing.

**Methods:**

A qualitative study was undertaken using an *exploratory policy framework* to guide the collection of in-depth interview data on GP surgeons’ training experiences. A purposive sample of GP surgeons currently practicing in rural BC and Alberta communities yielded interviews with 62 participants in person and an additional 8 by telephone. Interviews were audio recorded and transcribed then subjected to a *process analysis.*

**Results:**

Participants thematically identified *motivations* for acquiring advanced skills training, *resources* required (primarily in the area of solid mentorship), the most efficacious *context* for a training program (structured), and differences in mentorship between obstetricians and general surgeons.

**Conclusion:**

Mentors and role models were the most salient influencing factor in the trajectory of training for the participants in this study. Mentorship between specialists and generalists was constrained at times by inter-professional tensions and was accomplished more successfully within a curriculum-based, structured environment as opposed to a learner-responsive training environment.

## Introduction

Throughout much of rural Canada, surgical services have historically been the domain of General Practitioners (GPs) with enhanced skills in low-risk surgical procedures.^1^–^4^ These GP surgeons,* as a whole, represent an aging population, which has led to the rapid erosion of rural surgical programs over the last two decades.^6^,^7^ In western Canada in particular, prior to 2000 there were 76 communities with local surgical services provided by GP surgery and GP anesthesia teams. Twenty of these services were located in British Columbia (BC).^2^ Currently, only 15 of these communities continue to offer local surgical services.^8^

BC’s remaining and aspiring GP surgeons are beset by a number of challenges, including lack of collegial and health authority support, lack of regulatory structure, and lack of formal training avenues and continuing medical education (CME) opportunities.^7^ Formal training in GP surgery in Canada is currently at a standstill, although tentative steps are being taken in some jurisdictions to support an enhanced surgical skills program.

Where trainee GP surgeons have been successful in designing their own programs, they have often faced hostile learning environments or competition from specialist residents for surgical training positions.^9^ Moreover, this ‘custom-built,’ non-accredited training pathway presents credentialing and privileging challenges for health authorities and often makes GP surgeons’ skill sets non-portable between communities. GP surgeons attempting to learn a new procedure or maintain competence in an existing one must overcome the financial stress of leaving their community and practice, often for several months.^10^

The literature outlining training protocol for GP surgeons is scarce and qualitative literature describing the training experiences of GP surgeons is nonexistent. An American study by Deutchman et al. in 1995 indicates that GP surgeons trained in cesarean sections performed an average of 46 such operations during residency (ranging from 25 to 100),^11^ but this study falls far short of explaining the mechanisms for accessing such training for GPs. It also does not explain how GP surgeons are taught the crucial elements around the procedure, such as deciding when an emergency cesarean section is warranted.

A limited number of studies examining the training experiences of general surgeons exist. Doty et al.^12^ determined in a 2006 survey of rural general surgeons that exposure to a broad scope of procedures during residency was one of the most important factors in the decision to establish practice in a rural community. Positive training experiences also play a significant role in a student’s decision to pursue a career in surgery.^13^,^14^ Kemp, Zuckerman, and Finlayson^15^ found that American rural general surgeons can learn new procedures just as safely and effectively as their urban counterparts. However, there is no clear understanding of how rural surgeons are able to overcome the geographic, professional, and financial challenges associated with upgrading training, as the nature of rural surgery demands that surgeons be competent in a wide variety of procedures. Also, as generalists, GP surgeons rely heavily upon good communication and strong positive relationships with their specialist colleagues to provide expertise and guidance.^16^ In order to support any GP surgical training programs, therefore, it is critical that the training experiences of GP surgeons be understood in order to establish clear avenues for positive training experiences.

## Methods

### Overview and rationale of research design

This qualitative study was undertaken using an *exploratory policy framework* to guide the data collection and analysis of GP surgeons’ training experiences. The phenomenon of GP surgery is not well understood in the Canadian context and is only recently becoming an area of concern internationally. To develop knowledge of this field of practice, data collection consisted of in-depth interviews with GP surgeons in rural communities in BC and Alberta†, documentary analysis of policy and administrative records, and information requests from key contacts including educational program registrars.

### Sampling Plan

A purposive sample of GP surgeons currently practicing in rural BC and Alberta communities was used. Criteria for community selection included:

1. Presence of GP surgeons with advanced obstetrical skills;
2. Diversity in:
  - physical geography
  - degree of isolation
  - distance to referral centre, and
  - practice models (i.e. solo GP surgeons/GP surgeons working with specialists)

Based on these criteria, research was carried out in British Columbia in the communities of Revelstoke, Golden, Princeton, Vanderhoof, Burns Lake, Quesnel, 100 Mile House, Lillooet, Kitimat, Smithers, Bella Coola, Gibsons, Squamish, Creston, Fernie, Fort St. John, Fort Nelson, and Dawson Creek. Alberta communities included Peace River, Westlock, Grande Prairie, Hinton, Fairview, Edson, High River, and Pincher Creek.

Interviews were undertaken with 62 participants in person and an additional 8 by telephone. Potential participants were contacted by the project investigators by mail to solicit participation followed by a ‘snowball’ technique used to identify other practicing GP surgeons within the original or adjacent communities. As the project investigators have established research, professional, and research collaborative relationships with members of the study communities, recruitment was successfully accomplished with no potential interviewee declining to participate. Participation involved one in-depth interview and the optional review of the findings to assess their accuracy, relevance, and comprehensiveness.

### Data Collection

In-depth semi-structured qualitative interviews were done in person or by telephone with each participant. Questions and probes were informed by two pilot interviews that were undertaken with members of the target population. Example interview questions involved themes of education and training (“Describe the program you trained in”), relationships (“Do you have obstetricians practicing in your local community? If so, what is your relationship with your obstetrician colleagues?”), and practice experiences (“What are the benefits/challenges of rural practice?”). All interviews were audio-recorded with participants’ permission and transcribed.

### Data Analysis

Analysis of interviews was undertaken using a *process analysis* framework. We used flow charts to map the elements that comprise GP surgery training programs to convey the complexity and interdependence of program parts. To this end we considered the *resources*, *activities*, *benefits* and *impacts* of participants’ training experience, as well as their motivations for acquiring advanced skill sets for rural practice. The qualitative data analysis software program NVivo was used to aid in the analysis, specifically in regards to coding the data (attaching key words or tags to segments of text to permit retrieval; storing the data; linking the data; and writing commentary on the process of collection and analysis, or ‘memoing’).

## Results

Participants in this study expressed consistent themes in their descriptions of acquiring general surgical skills, underscored by the common motivation of responding to the needs of their community and enhancing personal competence. Their experiences are conveyed in the context of resources required, training activities, implications and challenges and the implications for rural practice. Taken together, these themes create a composite picture of the experiences of participants in this study and give rise to a discussion of the importance of mentorship in GP surgery training. Each theme is presented below.

### Theme 1 - Motivations for acquiring advanced skills training

Participants articulated an array of motivations for seeking out advanced skills training, including receiving encouragement from colleagues, responding to the needs of their community, enhancing their competence in the care they provide, and gaining professional satisfaction from a comprehensive slate of skills. Many trainees noted the importance of self-motivation to the success of their training. This included, but was not limited to, finding opportunities for training. For others, health human resource concerns were a prime motivation for acquiring advanced skills. As one interviewee expressed:

We were down to one obstetrician and there weren’t obstetrical locums around, so we were ending up with really horrible situations. One day I had a prolapsed cord and I got the South African ophthalmologist off the golf course to come and do a C-section, at which point I realized that I could do it better than they could. So then I decided to go away [for training]. It was sort of demand driven, because we didn’t have any obstetricians.

### Theme 2 - The Importance of mentors, role models and funding

Mentors and role models were the most salient influencing factor in the trajectory of training for the participants in this study. As many trainees noted, however, mentors were usually specialists, such as general surgeons or obstetricians. The lack of visible GP surgery role models was noted by most of the study participants:

These opportunities are not very visible you know, family practice medical studies are presented with career choices of specialization and there is very little on their radar screen that offers the third way which is a primary care with procedural skills which is rural medicine. I think there is a lot to be done in increasing the visibility of that option but you can’t do it unless the programs are going to be accessible for training.

Characteristics common to positive mentors were recognized by many of the trainees, including positive regard and encouragement, respect for the field of GP surgery, and an understanding of the unique demands of general practice in a rural community. Likewise, several respondents related stories of positive feedback they received and how important that was, not only to their training trajectory but also to their motivation to complete training:

I phoned the surgeon I trained with and I said this terrible thing had happened and I think that I should not do GP surgery anymore. He said, ‘Don’t worry. That’s your complication of a lifetime and it will never happen again.’ He told me to keep going.

Good mentors, many observed, were often those who had trained as a general practitioner before specializing. Although rare, these mentors were noted for remaining ‘sensitive to the needs of GPs’. Other respondents reflected positively on the openness of their mentors in allowing the trainee to participate in or undertake procedures. This openness, combined with imbuing a sense of confidence in the trainee, went a long way for those who were fortunate enough to be the recipient. As one trainee noted:

The principal preceptor was a very experienced, gentle, patient obstetrician-gynecologist who had tons of experience. He basically said, ‘You know what? Just start.’ And he gave me the knife and the first procedure we ever did together was a set of twins that was double breech. And I’ll never forget that day. It was one of the most exciting days in my medical life. He just said, ‘Start cutting.’

In addition to teaching technical proficiency in discrete surgical skills, good mentors taught trainees more generally how to “think like a surgeon,” to anticipate when surgery was necessary or unnecessary and when the procedure required a higher level of skill and infrastructure than they could provide.

For most participants, the expense of advanced skills training was funded either by continuing medical education funds or by the trainees themselves. Most participants who trained in British Columbia were funded through the Rural Education Action Plan (REAP) program. Prior to this program, trainees had to self-fund their surgical skill development, sometimes with help from local hospitals. As one participant noted,

[When I trained] there was no REAP program. There was nothing, right. The hospital gave me one thousand dollars to go train, and I had to sign a contract stating that I would be available for the next five years.

Although most participants acknowledged REAP’s contribution in facilitating their training, they also noted a lack of direction and guidance on how to secure their training, where to access training, or what kind of training they should do. Alternatively, Alberta trainees expressed that they were self-funded in their training as there was no program equivalent to REAP in that province at the time of their education.

### Theme 3 - GP surgery training in British Columbia, Alberta and internationally

The GP surgeons interviewed in this study trained in British Columbia or Alberta, while some acquired their skills internationally, namely in South Africa. Among the BC and Alberta-trained participants, there were notable differences between the training experiences in each province, and further differences between the experiences of those who trained in Alberta prior to and after 2000.

The program in BC was described as learner-responsive, being “on individual initiative”. In BC, practitioners had to secure mentorship and determine the procedures in which they wanted proficiency, without the aid of an organized curriculum or planning guide. This lack of structure led to discomfort, uncertainty, and ineffective planning for many of the participants. As one noted,

There was no precedent for what I… what was I supposed to do? And where? And how? So I really felt like I kind of cobbled it all together myself, and it kind of worked and it kind of didn’t work.

BC training experiences were also *location-specific*; that is, trainees pursued skills that would be useful to their community’s population. One participant, however, noted the non-portability of such training, saying, “This seems to fly in the face of the kind of mobility that the rest of the country is trying to encourage in people acquiring these skill sets”. Several trainees spoke of how they intuitively structured their training according to community need in the absence of guidelines:

I thought I would go to a community of about 5–10,000 people and I got some OR lists from a few communities that fit that size description and then looked to see what kind of procedures were done quite often. Then I designed a skill set that I thought would be useful and then looked at communities that I thought I could do that training in.

Due to the vagaries of rural training and the need to take advantage of the opportunities presented, however, several trainees noted that their plans changed once they were at a training site, usually due to the lack of opportunity for learning that is concomitant to low-volume settings.

The program in Alberta was more structured than that in British Columbia and required six months immersion in obstetrical surgery and six months in general surgery. There were also temporal differences in the experiences of those who trained prior to 2000 compared to those who trained after 2000. Prior to 2000, the three general surgeons who staffed the program had a background of training and practice in small towns in a time of truly-full scope general practice and expected their trainees to work in the same way. Upon retiring, these general surgeons were replaced with general surgeons who had trained within a more subspecialized culture and manifest a more proprietary relationship towards surgery; not unlike the evolution of general surgery in most of Canada. Participants noted that this philosophical difference resulted in reduced exposure to procedural training and lack of support from the preceptors:

I think it’s the third guy that worked there who was kind of dragged kicking and screaming into helping me out. And he hardly ever preceptored me, was more ‘towing the party line’ when it came to general surgery. He let me do a couple of appendices but at one point when he was consulting for a patient he didn’t even call me in, which was exceedingly irritating because he got paid to do the program.

One limitation of the Alberta program noted was the limited number of applicants (two) accepted to train every year. However, mitigating this was that low numbers of trainees within the context of large volumes led to the lack of competition for cases among those who were training, and thus provided increased experience:

It was great. I had a great time, I was the only resident in [the program] and I got my pick of what I wanted to do, whether it was surgery or medicine or obstetrics or urology. I was the resident so I got to do everything I wanted.

Generally, those who trained through the Alberta program expressed a general sense of confidence and competence in procedural care in rural communities and the capacity to recognize which procedures needed to be referred. However for three trainees we interviewed, undertaking GP surgery training led them to continue to specialize in surgery due to their feeling that they did not have enough training after one year to practice on their own.

Many GP surgeons we interviewed had trained or refreshed their skills in foreign countries, namely South Africa. Others expressed very positive experiences of working with or being mentored by South African-trained GP surgeons:

I think he gave me the best training because he worked as a GP surgeon himself in South Africa first, so he had an idea about risk management and he did a lot of teaching with me about how to decide who to operate on and who not to operate on. And that was probably the best training.

Many trainees recognized the foundational contributions their South African colleagues make to ‘fill the gap’ and sustain rural GP surgery in Canada in the absence of a robust Canadian training system.

Most participants noted a difference in obstetrical training versus training involving general surgery skills, finding that obstetricians were generally more amenable to having GPs learn enhanced skills. One participant described the obstetrical internship, emphasizing the difference in approach between obstetrics and general surgery:

I did a bunch of laparoscopic cases Thursday mornings at [the hospital] with a preceptor. One day after a few months he said, ‘You’re fine. Go home and do laparoscopies.’ And I said, ‘Okay, thanks.’ And that was how that was done [with the gynecologists]. It’s almost impossible to do that with general surgery.

General surgeons, on the other hand, were described as adhering to the guideline, “Thou shalt not enter the peritoneal cavity if you’re a GP”, and were generally less forthcoming with support. This was recognized by all of the trainees interviewed. One tangible area of conflict that arose for many of the trainees was competition with general surgery residents for OR time and mentorship. Many noted that mentors gave priority to their general surgery residents for surgical cases.

The tenor of training experience and environment is intimately tied to the accreditation of the trainee. Most trainees described an informal approach to accreditation by their preceptors. One noted, “I went to [a large hospital] and spent time there and came back with a letter saying, ‘This guy’s probably safe to do C-sections,’ and started doing C-sections”. Likewise, accreditation occurred within the trainees’ community if there were more experienced practitioners willing to attest to their competence. In other cases, however, participants conveyed how uncomfortable preceptors were in assessing their competence due to the potential legal liabilities. One described how her preceptors resisted accrediting her training:

So even though I go over and I work with them and I do a few appendixes, they’re very clear that they’re not telling me that I’m safe to do appendixes. I’ve run into that even with the colonoscopy issue. They actually put it in writing they felt so strongly about it and sent me a letter to say that this in no way validates you to do colonoscopies.

### Theme 4 - Training implications

Beyond the clearly articulated benefits around increased skills allowing more comprehensive care to their communities, many of the interview participants we spoke with recognized attendant benefits to their training, including the increase in overall competence and confidence within their community of practice. One trainee noted,

I’ve always said when I finish my training that even if I never did things surgically, the training and experience would be valuable to regular good old-fashioned general practice with emergency privileges. So even if I couldn’t do surgery, it would certainly help with patient stabilization, assessment and all those types of things.

Others noted the increased level of comfort for residents doing procedures like sewing up extensive lacerations. One noted, “It gives them more confidence to do the things that rural physicians have to do sort of unsupervised”.

The predominant negative experiences of training for participants in this study focused on difficult relationships with mentors. These relationships were characterized by a lack of support, lack of opportunities to learn new skills under their supervision, disparaging comments, and in some cases outright hostility. These training experiences were formative for many of the study participants, in that it undermined their self-confidence. In the extreme, some trainees were told outright that surgery in rural areas would lead to disastrous consequences:

[There was] a lot of criticism, a lot of questioning. ‘You’re crazy, you shouldn’t be doing this, you shouldn’t be pursuing this in your career,’ and just a really negative [approach]. You know, he would say things like, ‘You’re going to be in trouble because when you do C-sections in rural areas you know, most of them are going to end up needing emergency hysterectomies post…’ Very stressful when you’re in a learning situation.

Larger hospital environments were sometimes suspicious of enhanced skills in rural practice. This had significant consequences for some trainees. As one respondent noted, “They convinced [one trainee] that he was wasted being a doc in a rural community.” The trainee, who was learning GP anesthesia skills, ended up training to be a specialist.

Interviewees described barriers to training within the context of the current programs, including a lack of structure and guidance in the BC program, lack of professional support, and competing priorities among all the other responsibilities rural physicians undertake. Additionally, several trainees noted that although funding was now forthcoming through the BC REAP program, time away from the community was an insurmountable challenge to training. As one summarized,

I think a barrier to GPs not becoming GP surgeons is [that] it costs money to go and train them, money and time. [Then there are] all the other things that family doctors have to be able to do in rural Canada, such as emergency work, chronic care, palliative care… now to add other expectations or skill sets may be difficult.

Others spoke of the tenuous future of rural surgery generally, particularly with health human resource shortages in nursing. One expressed concern about the long-term utility of his advanced skills training:

For me to commit to getting more skills and training, I have to know that the hospital is committed to obstetrics as well. And in the three years that I’ve been here they haven’t come through. So it kind of leaves me thinking, ‘How much more should I put in if, you know, they’re not going to come up with the nursing staff to really make this happen?’

### Theme 5 - Implications for rural practice

Nearly all of the participants in this study commented on the implications of the lack of a robust training program for GP surgeons in Canada. One trainee, who had returned to his home community post-training, noted the challenges in recruiting surgical colleagues:

So we are actively recruiting. The problem is there is no one around. No Canadian-trained GP surgeons, for sure, and overseas has dried up. The South African physicians aren’t coming as often as they were, and when they are coming they don’t have the skill sets that they used to have. We’re stuck, you know. Our service is in jeopardy of closing completely within the next six months to a year.

Despite challenges to training programs, leading to threats to the sustainability of the profession, some respondents had a positive vision for the future. One noted:

I think there is a real renaissance in rural medicine. I think that people see it as a viable career option now, where people didn’t before.

Others expressed their confidence in the altruism of the residents interested in rural medicine and training in advanced skills within a context of new graduates pursing more lucrative options, such as walk-in clinics. Most study participants, however, noted the tenuousness of the rural surgical services, services that are threatened if practitioners decide to retire or move.

## Discussion

The resilience of GP Surgery programs in small rural communities is remarkable, considering the training experiences of most of the individuals interviewed in this study. The issue of mentorship in medical education is an emergent one, that previously took a back-seat to more pragmatic concerns such as the uneven distribution of physicians, often interpreted as a shortage.^17^ Within medical education, mentoring is broadly defined as a process of extending supportive wisdom, guidance, and reflection in an *informal* way on the part of the mentor. It is regarded as a key component of ensuring recruitment and retention,^18^ in role-modeling,^19^ introducing practical training, and increasing confidence,^20^ improving outcomes,^21^ and encouraging trainees to take up surgical training.^22^ The literature, however, neglects to consider the negative implications of both the lack of mentorship and ineffectual mentorship, both of which can lead to significant psychological damage on the part of the trainee and even contribute to their abandonment of the pursuit of training.

In their paper “Mentoring and surgical training: a time for reflection!”, Memon and Memon^23^ point out the dilemma in medical education of espousing the benefits of mentorship while at the same time providing little support for anything outside the auspices of formal training. This dilemma is accentuated in the case of GP surgeon trainees, as mentorship - formal or informal - is currently the only route to achieving their professional goals due to the lack of formal training programs. As these practitioners are rural by definition, their professional isolation is magnified by a lack of peer support and geographic isolation from others of similar practice. Inherent challenges ascribed to mentors include lack of time^24^ and the generally non-profitable nature of the relationship.^23^ Indeed, though incentives are occasionally discussed, the key element of remuneration is seemingly avoided in the emerging literature.

Without a system-level recognition of the importance of those who guide new physicians in acquiring enhanced skills, and a framework that both supports and remunerates mentors for their time and skill, we are relying on tenuous supports in preparing new practitioners to contribute to the health needs of rural Canadians. The instability of the situation, further entrenched by inter-professional challenges, urgently needs to be addressed.

## Conclusion

Small, isolated rural communities (> 5000) have needed, and will continue to need, local surgical services in order to provide safe care to their populations. General surgeons have historically provided training support for general practitioners with the interest and commitment to provide these services. The experiences of Canadian trained GP surgeons interviewed suggest that contemporary training opportunities and mentorship are scarce, due in large part to the sub-specialization of general surgery and the retirement of existing rural general surgeon mentors. To ensure that the health needs of rural residents are met, key players including GP surgeons, general surgeons, obstetricians, academic leaders and rural health services planners need to work collaboratively to effect sustainable solutions. This crisis in rural surgical services is growing and needs to be addressed.

## Footnotes

* Typically, GP surgeons support programs in rural communities with populations of 5–15,000, while mixed models of GP surgeons and specialist surgeons support communities of 15–25,000. Procedural scopes of practice for GP surgeons vary based on training experience and can consist of providing solely cesarean sections or can include a range of other procedures such as appendectomy, endoscopy, hand surgery, herniorrhaphy, tonsillectomy, laparoscopic tubal ligation, dilation and curettage (D&C), and cesarean section. An analysis of the outcomes of GP surgery has indicated equal quality of care to what is offered in larger centers.^5^

† Our working definition of ‘rural community’ is a community with a population of under 10,000 that is more than a one-hour commute from larger metropolitan centres [Statistics Canada, 2010. Available at: http://www.statcan.gc.ca/pub/21-006-x/21-006-x2001003-eng.pdf November 20 ].
